# Public values and plurality in health priority setting: What to do when people disagree and why we should care about reasons as well as choices

**DOI:** 10.1016/j.socscimed.2021.113892

**Published:** 2021-05

**Authors:** Rachel Baker, Helen Mason, Neil McHugh, Cam Donaldson

**Affiliations:** Yunus Centre for Social Business and Health, Glasgow Caledonian University, Scotland, UK

**Keywords:** Public values, Plurality, Priority setting, Q methodology, Incompletely theorized agreements, Health economics, Empirical ethics, Public deliberation, Preference elicitation

## Abstract

**Context:**

‘What does ‘The Public’ think?’ is a question often posed by researchers and policy makers, and public values are regularly invoked to justify policy decisions. Over time there has been a participatory turn in the social and health sciences, including health technology assessment and priority setting in health, towards citizen participation such that public policies reflect public values. It is one thing to agree that public values are important, however, and another to agree on how public values should be elicited, deliberated upon and integrated into decision-making. Surveys of public values rarely deliver unanimity, and preference heterogeneity, or *plurality*, is to be expected.

**Methods:**

This paper examines the role of public values in health policy and how to elicit, analyse, and present values, in the face of plurality. We delineate the strengths and weaknesses of aggregative and deliberative methods before setting out a new empirical framework, drawing on Sunstein's Incompletely Theorised Agreements, based on three levels: principles, policies and patients. The framework is illustrated using a recognised policy dilemma – the provision of high cost, limited-effect medicines intended to extend life for people with terminal illnesses.

**Findings:**

Application of the multi-level framework to public values permits transparent consideration of plurality, including analysis of coherence and consensus, in a way that offers routes to policy recommendations that are based on public values and justified in those terms.

**Conclusions:**

Using the new framework and eliciting quantitative and qualitative data across levels of abstraction has the potential to inform policy recommendations grounded in public values, where values are plural. This is not to suggest that one solution will magically emerge, but rather that choices between policies can be explicitly justified in relation to the properties of public values, and a much clearer understanding of (in)consistencies and areas of consensus.

## Highlights

• Public values are important for priority setting in health.
• Public values are plural but we do not know how to account for plurality in policy.
• Counting, coherence and consensus are relevant to resolving value plurality.
• This paper presents a new empirical framework for analysis of public values.
• The framework has potential to account for value plurality in health policy making.

## 1 Introduction

‘What does ‘The Public’ think?’ is a question often posed by researchers, policy makers, politicians and journalists. Public values are invoked, often without evidence, to justify policy decisions. Over time there has been a participatory turn in the social and health sciences, and in public sector decision-making, with requirements for public involvement written into the institutions and structures of health systems (NHS England, 2019; NHS England/Public Particiption Team, 2017; Canadian Institutes of Health Research, 2020). Public and patient involvement (PPI) and engagement (PPIE) is now a formal requirement of many health research funding bodies such as the National Institute for Health Research (NIHR) in the UK (NIHR, 2014). This is, arguably, a good thing. Involving the public in decisions about public resources is democratic – as citizens we should be able to participate in decisions that affect us. More instrumentally, we might argue that decisions that take account of public values and preferences are more intelligent and will lead to better outcomes in terms of social welfare. This is a standard assumption of cost benefit and social welfare analyses in economics, but the instrumental value of public involvement in decisions about public resources is an assertion also made more widely (Stevenson, 2016).

Health priority setting is the process by which options are evaluated and resources allocated, determining who gets what (Mitton and Donaldson, 2004; Weale et al., 2016). Explicit forms of health priority setting usually centre on appraisal of the costs and benefits of different courses of action. But, priority setting in health is a complex, multi-criteria decision process and, despite recent advances, (health) economic evaluation has, in the past and also recently, struggled to account for broader considerations in relation to equity and fair distribution of resources (Mitton et al., 2019; Hadorn, 1991; Cookson et al., 2017; NICE, 2009). Even when faced with the same economic evidence, reasonable people might disagree about how best to allocate resources (Daniels, 2000). In such situations, where collective resources and distributional justice are at stake, public values are increasingly included as one part of health priority setting practices and frameworks (Mitton et al., 2011; Abelson et al., 2013; Baltussen et al., 2017).

It is one thing to agree that public values are important, however, and another to agree on how public values should be elicited, deliberated upon and integrated into decision-making. In this paper we focus on the first two of these issues, elicitation and deliberation, and raise some consequent issues for decision making in the discussion section. Typically, health economists elicit values and preferences through surveys of representative population samples (Shah et al., 2015; Pinto-Prades et al., 2014; Pennington et al., 2015; Rowen et al., 2016; Lancsar et al., 2011); bioethicists place emphasis on the quality and coherence of arguments (Norheim, 2016; Fourie et al., 2012; Biron et al., 2012) and might question the relevance of participation by (ill-informed) publics; political scientists and advocates of public deliberation have designed processes to surface public values in a way that can inform policy (Abelson et al., 2013; Escobar, 2017; Fleck et al., 2012). But in all such surveys and processes disagreements, preference heterogeneity or *plurality*, are to be expected (Daniels et al., 2012).

Plurality presents challenges for policy development that seeks to take account of public values. How can one policy take account of multiple, competing perspectives? Different disciplines take different approaches to account for plurality in public values in a way that might lead to policy recommendations, placing different emphasis on *counting* (majoritarianism, strength of preference), *coherence* (logical consistency and moral argument) and finding *consensus* (deliberative, talk-centred methods). But generally there are two routes researchers employ to address plurality; aggregation or deliberation (Gutmann and Thompson, 2004).

In this paper, we unpack the approaches to public values in priority setting before bringing together the strengths of aggregation and deliberation in an interdisciplinary framework, influenced by Sunstein's *Incompletely Theorized Agreeements* (Sunstein, 1995, 1998). This multi-level framework delineates high-level values from mid-level policies, norms or rules, and further, from the particulars of cases or specific decisions. At essence this renders visible the *nature* of plurality in public values and permits transparent consideration of coherence and consensus, in a way that provides options for policy recommendations. Finally, placing the framework into a real policy context - the provision of expensive medicines of limited effectiveness for people with terminal illnesses - we provide examples of methods through which the framework might be used to structure empirical research into public values.

## 2 Defining terms: public(s), value(s) and priority setting

Before setting out the framework, some key terms – ‘public’, ‘public values’ and ‘priority setting’ – warrant brief explanation.

### 2.1 Public(s)

There are many potential *publics* that can be constructed for policy making (Escobar, 2017; Stewart, 2016). We use the term here to point to the role of citizens and distinguish from the values we might elicit from patients or users of health services. Patient and public involvement (PPI) and engagement (PPIE) are catch-all terms commonly used in health research. ‘The Public’ is clearly the larger group of which patients are a subset, but there is emerging consensus in the academic literature that *patient* and *public*, rather than describing categories of people, should be seen as roles that people (are asked to) occupy, roles with particular expectations and claims.

Fredricksson and Tritter (2017) helpfully disentangle the Ps in PPI, drawing up ideal types for patients and publics (Fredriksson and Tritter, 2017). Patients are health services users with experiential knowledge and a sectional (rather than societal) interest in the health and wellbeing of a particular interest group. The role of citizens is to focus on the wellbeing of the general public, to adopt collective perspectives and to represent societal rather than sectional interests. As disinterested members of the public, citizens are expected to take a more detached role (Lehoux et al., 2012) that does not entail particular knowledge, personal interest or advocacy, but reasonable judgments about the value placed on different states of the world (with respect to the distribution of health resources) from behind a Rawlsian ‘veil of ignorance’.

There are a number of important discussions about seeking ‘ordinary citizens’ for public engagement roles (Martin, 2008), the so-called lay paradox (Ives et al., 2013) and the “ontological lightness” of citizens in this context (Lehoux et al., 2012). These are beyond the scope of this paper and here we make use of a minimal definition of public in terms of *the role of citizens* and the societal perspective that implies.

### 2.2 Public Value(s)

For purposes of this discussion, we allow ‘public values’ to remain quite a broad term, which reflects its use in health priority setting literature and policy documents (in which publications we see reference to *social* or *societal* as well as *public* values). Following Clark and Weale, we distinguish public values from pure moral values in that public (or social) values are based on the values of a particular society at a particular time (Clark and Weale, 2012).

The term values has at least two distinct connotations: i) value, used in the economics sense to mean measurable strength of preference for a good or service, expressed through trade-offs or sacrifice; and ii) values as higher level principles, moral or socio-cultural standards that people hold as good or right. In this sense values are normative commitments that might motivate or justify different courses of action. For example, the choice ‘I would prefer hypothetical patient A to benefit from scarce resources over patient B’ is a judgement implying more *value* (in the economics sense) is placed on treatment of patient A; it is not a moral principle. A health maximising reason for that choice (because overall health gain is greater if we invest in treatment A) reflects a commitment to the principle that we ought to make decisions that maximise health. Both i) and ii) are important components of the framework ultimately proposed.

It is worth noting that ‘values’ and ‘principles’ are terms often used quite interchangeably in the priority setting literature and beyond. There are semantic distinctions and different definitions not only in ethics and economics as highlighted here, but also sociology, psychology and in the ways in which policy communities might use the term ‘values’ (Datta Burton et al., 2021; Jain et al., 2019). NICE produced a ‘Social Value Judgements’ document in 2008 (NICE, 2008). Although the document title uses the terms value and judgements, it refers throughout to principles. Values might be seen as more fundamental, or foundational, than principles, but the level of abstraction is not always clear. For example in their work on social values in health priority setting Clark and Weale (2012) include fundamental values such as solidarity and autonomy alongside cost effectiveness (which they note is perhaps a balancing of other values).

It is beyond the scope of this paper to resolve discussions about what constitutes values versus principles, but fortunately the framework we propose below does not insist on a particular reading of such terms; only that these are both of a higher order than choices or judgements. Both imply claims about what is right, fair, or what things should matter.

### 2.3 Priority setting and resource allocation

‘Priority setting’ is a term used commonly in health economics and health technology assessment (HTA). It has largely replaced the word ‘rationing’ and its connotations of wartime shortages of basic goods, although they mean broadly the same thing. Priority setting implies decisions in relation to who gets what (e.g. services and technologies), as a consequence of which there will be winners and losers. In the context of a fixed budget and an existing allocation of resources, if all operational efficiency gains have already been exploited, a decision to invest in X means disinvesting in Y. Much attention is given to HTA and decisions about the adoption of new technologies in the literature on priority setting in health (Bryan et al., 2014). However, priority setting is wider than HTA (Mitton et al., 2019) and there is a range of potential settings in which priority setting takes place, as described by Weale et al. (2016):

… Priority setting in health care does not simply take place in the setting of HTA agencies, however. Cabinets, government departments, health care agencies and local authorities all have a role in priority setting through their routine decisions on resource and budgetary allocations, decisions on capital spending, price negotiations with manufacturers on pharmaceutical products and medical devices as well as investments in the training of medical and para-medical staff. Courts play a role in adjudicating the extent to which some of these decisions, when contested by plaintiffs, conform to administrative, constitutional or international law. Hospitals and insurance agencies make decisions on which services to provide and to whom. And individual physicians are inevitably involved in making decisions on health priorities when they make treatment decisions with their patients. From boardroom to bedside the determination of priorities is implicit in an organized health care system. (Weale et al., 2016) p 737.

In this paper, we are concerned with how we can evidence and analyse plurality in public values to better inform priority setting in relation to policies dictating the provision of care and services (noting more broadly that public values can be put to other uses, such as to inform marketing or political campaigns). We distinguish this from clinical decisions about treatment of individual patients and from individual treatment requests or judicial appeals which we see as exceptions and challenges to public policy (although clearly these individual decisions influence resource allocation).

### 2.4 Plurality and policy: aggregation versus deliberation

If we take the view that research should have relevance (or going further, societal impact) it is not sufficient for researchers to report and describe plurality in public values without reference to possible policy recommendations or other societal purpose. The two main routes to resolving plurality for policy recommendations are aggregation and deliberation. Gutmann and Thompson (2004) summarise the strengths and limitations of aggregation and deliberation before drawing their conclusions in support of the latter. In the following subsections we draw on Gutmann and Thompson, noting examples of aggregative and deliberative approaches in studies of public values in health priority setting, with a view to outlining a single framework that combines the strengths of both.

### 2.5 Aggregation

Aggregative approaches tend to elicit, and sum, values from representative, general public samples using methods that incorporate trade-offs; the limits of our willingness to make sacrifices in return for a good, or changed state of the world, represents the value we place on it.

Stated preference methods such as willingness to pay (WTP), discrete choice experiments (DCE), or person trade off (PTO) are used in health and welfare economics to elicit preferences and values. Surveys of this kind do not challenge citizens’ preferences - aggregative approaches generally take public values as given and do not ask question about the (quality of) underlying reasons. This might be seen as a strength to some analysts who would like to see *unfettered* preferences, so far as it is possible, to avoid researchers leading or laundering values. Because the resolution of disagreement can be represented numerically, citizens can see how decisions are made. Such transparency is important for those with minority preferences whose values might not be respected by policies based on majority rules.

Responses tend to be equally weighted in aggregation. Hence participants’ voices are equally heard. Small, selective or biased samples, of the sort used in deliberative exercises, will lack legitimacy because they distort the distribution of population views and preferences the sample has been selected to represent. Large samples also generate diversity, and inclusion of diverse perspectives is likely to lead to better outcomes (Stevenson, 2016).

But perhaps the most compelling argument for aggregative approaches is the ostensibly straightforward resolution of plurality: aggregation produces determinate results, an answer emerges. More problematically, plurality is hidden behind averages and the normative basis of different types of aggregation is rarely discussed (Devlin et al., 2017; Schneider, 2021). Although transparency is listed as a strength, as aggregative methods become more complex they also become more opaque to the non-specialist (few non-economists would claim detailed knowledge of the cost benefit analyses underpinning transport safety policy, for example). Aggregation pays no attention to the reasoning behind public values or the quality of arguments, nor to the fact that preferences might change after deliberation. The possibility of ill-informed or inconsistent preferences is not attended to, with all responses treated as equally valid. Furthermore, where one option wins out by only a small margin, aggregation is silent with respect to the justification or practical implications of a large minority being overruled. Beyond *observing* and making visible the so-called heterogeneity in public values, it is not clear what the health economist would or could recommend other than the policy implied by aggregation of preferences.

### 2.6 Deliberation

Public deliberation takes different forms but can be summed up as talk-centred approaches with ‘ordinary people’ aimed at finding collective solutions to social problems (Blacksher et al., 2012). The use of deliberation is growing (Abelson et al., 2013; Mitton et al., 2009), as exemplified in well-known health care institutions; for example, in the UK, NICE Citizens Council between 2002 and 2015; and in Canada the Citizens' Reference Panel on Health Technologies [CRPHT] established in 2008 in Ontario. There is a range of approaches to public deliberation, collectively referred to as ‘mini-publics’, including citizens' panels, citizens' juries, deliberative polls, planning cells, and forms of participatory budgeting (Escobar and Elstub, 2017). In an often-cited essay, ‘What is public deliberation?’ Blacksher et al. (2012) propose a minimum definition with three key elements:

“(1) the provision of balanced, factual information that improves participants' knowledge of the issue; (2) () the inclusion of diverse perspectives to counter the well-documented tendency of better educated and wealthier citizens to participate disproportionately in deliberative opportunities and to identify points of view and conflicting interests that might otherwise go untapped; and (3) () the opportunity to reflect on and discuss freely a wide spectrum of viewpoints and to challenge and test competing moral claims.” p16

On this definition, deliberation requires the location of common ground, not necessarily consensus or unanimity, but an agreed set of outputs that can be used for policy decisions (Blacksher et al., 2012).

The strengths of public deliberation are clear: it produces solutions and policy recommendations based on the reasoned exchange of views between citizens, after consideration of balanced information about a social problem. Resources are committed to provide time and conditions for a high-quality discussion, respectful questioning of views, listening and reason-giving, drawing on more abstract values and principles to justify positions. Participants should be open to the possibility of changing their minds in response to stronger arguments. By finding areas of common ground, proposals can be made that people with diverse views can live with.

Public deliberation is also contentious and criticised because of the size and representativeness of groups selected for deliberation and the (likelihood of) representation of diverse viewpoints. Parkinson (2003) raises a number of legitimacy problems with deliberative democracy focusing on the issue of scale. For deliberation to satisfy the qualities that recommend it, i.e. listening, reason-giving, common ground and adjustment of positions on the basis of better reasons, it must take place in small groups. The greater the number of voices in a room, the less likely everyone will be heard and the exchange will be well reasoned. But the fact that deliberation must happen in small groups by necessity, raises questions of selection bias, diversity and representativeness – not everyone can take part (Parkinson, 2003). Although many mini-publics call for randomly sampled, non-partisan citizens for public deliberation, representation of diverse viewpoints in any one group is left to chance.

More practically, and setting aside issues of scale, clear accounts of how deliberative groups reach consensus or agree collective recommendations are lacking. In a review of public deliberation in health policy and bioethics, Abelson et al. (2013) found that, although published studies adhere quite well to the minimum definition offered by Blacksher et al. (2012), there was ambiguity in relation to how representation and diversity are achieved, and a lack of clarity around value-based reasoning and “what is required for value-based reasoning that produces collective judgments to occur” (Abelson et al., 2013) p12. For legitimate resolution of disagreement, transparency is important, particularly about how recommendations were agreed, how views shifted, and which reasons carried force.

## 3 How to cope with plurality in public values for policy?

The discussion of aggregation and deliberation thus far highlights that given the strengths and weaknesses of each, neither approach, used in isolation, is sufficient if we seek to respect plurality in policy recommendations. So, can they somehow be combined in a way that builds on respective strengths whilst overcoming their weaknesses?

### 3.1 Disciplines, public values and plurality

Academic disciplines place differential importance on counting, coherence and consensus when it comes to resolving plurality. Table 1 sets out the ways in which economics, ethics and political science conceptualise and elicit public values (column 1); how plurality is addressed (column 2); and disciplinary emphasis on counting, coherence and consensus in resolving plurality. This is illustrative and selective, other disciplines such as law or sociology could have been included but we have selected those that would most likely seek to make (substantive rather than procedural) recommendations about priority setting in health taking account of scarcity. It is also worth noting that these caricatures do not represent the diversity and nuance that exist nor highlight examples of interdisciplinary work (Gibson et al., 2016). Nonetheless our broad characterisations help illustrate some of the key issues and competing perspectives at play in resolving plurality.

**Table 1:** Approach to public values and plurality by discipline – a simplification.

Discipline	How are public values and priority setting in health conceptualized/elicited?	How is preference plurality in public values addressed?	What emphasis is placed on i) counting ii) coherence (consistency) and iii) consensus?
Health Economics (and more broadly welfare/public economics)	Preferences are elicited using methods that present trade-offs (money/time/lives/health) to measure the value of health and health care and preferences over distribution of resources. Mixed methods work is still relatively uncommon in economics though interest is increasing. Choices are important and should be informed; higher-level reasons for choices might be seen as irrelevant by some economists.	Individual preferences are aggregated to estimate social (public) value. Some analysts might look at distribution of preferences, otherwise means/medians are likely to hide disagreement and difference. Extreme values might be excluded from analysis (outliers that are seen to unduly affect the mean value).	*Counting* is important through aggregation of large representative samples in stated preference studies *Coherence* is not considered. *Consistency* is considered in terms of irrational responses. Questions might be included to test rationality by repeating questions or presenting choices that are strictly dominated by alternatives. Inconsistent, responses might be excluded.
Ethics (and more broadly branches of moral and political philosophy)	Some ethicists would reject public values as irrelevant to good decisions. Public values can be reprehensible or ill-informed and normative claims cannot be based on public opinion. Approaches to procedural justice in health care priority setting have been influential (. In this context public values might be relevant in terms of the composition of a committee, acceptability of reasons (e.g. determining what is relevant), decisions and the right to appeal decisions.	Ethical analysis would consider the range of relevant viewpoints or stakes in the issue in terms of the strength of claims, arguments and reasons. Studies of empirical ethics might include public views or values as relevant empirical data.	Ethical analysis places emphasis on *coherence* of arguments, including logical consistency. *Counting* and *consensus* in public values might be relevant information for acceptability of recommendations.
Political Science (and particularly deliberative democratic approaches)	Public values are expressed through electoral systems and citizens' votes. Approaches to participatory, deliberative democracy, such as mini publics (such as citizens' assemblies, juries) gather citizens together. There is emphasis on quality of communication, interaction and informed participation. Sometimes deliberative events include polls and counts.	Typically the politician/party with the most votes gains majority and public values influence policies indirectly through constituency representation and/or lobbying. Mini publics tend to be randomly sampled in order to be representative. Plurality of views might occur by random chance; .	Representative electoral systems rely on vote *counting*. Some mini publics will include polls or votes but usually only small numbers. Some administrative (cross party) committees in government will aim for *consensus* and *coherent* arguments should carry force. Mini publics sometimes, but not always, aim for *consensus* (or a super majority of 60–80% agreement on a decision). Emphasis is placed on finding common ground. Through reason giving more coherent arguments should prevail.

It seems clear that *counting* and measuring values is important. The extent and intensity of preferences in a population, as indicated by magnitude of trade-offs members of the public are willing to make to achieve policy goals, is important. However, by focusing only on what is common (as quantitative estimates often do), we are deaf to whether arguments are *coherent*. Commonly held values might be logically inconsistent, or call on irrelevant principles and arguments. If coherence is an important basis for policy, which seems reasonable, then inconsistent public values or values based on irrelevant arguments are problematic.

Yet coherence as the sole criterion to resolve plurality is also insufficient. If more than one coherent public view on a subject exists, how can a single policy recommendation be derived?

Although deliberation is often concerned with seeking *consensus* about recommendations among people with different perspectives, consensus is also insufficient on its own, since we might find agreement on things that do not withstand critique in relation to their coherence.

If counting, coherence and consensus are each insufficient, but simultaneously important, then the way forward in accounting for plurality may be to explore the possibility of an integrated framework that allows examination of all ‘three Cs’ in empirical study and consequent policy recommendations.

### 3.2 Counting, coherence and consensus in an interdisciplinary framework

We propose an interdisciplinary, empirical framework, drawing on Sunstein's Incompletely Theorized Agreements (ITA) (Sunstein, 1995, 1998). It is offered as a means to combine the strengths of aggregative and deliberative approaches and to connect qualitative and quantitative methods, commonly used in each approach, in a unified empirical framework. In previous research, we identified and described public values in relation to provision of high-cost, life extending medicines for people with terminal illnesses (McHugh et al., 2015). We measured support for the three competing viewpoints we had identified and found, in brief, that:

i) survey respondents were roughly evenly split across two dominant viewpoints, with a third view supported by roughly 10% and
ii) the most common viewpoint did not consider the budget constraint, which we considered highly relevant (Mason et al., 2018).

Thus, for researchers hoping to draw recommendations from our findings, majoritarianism did not present a clear answer and we concluded that there was a need to (not only report but also) critique public views in terms of their coherence and completeness. Reflecting on our own findings and on related papers (Shah et al., 2018), we were looking for a route forward for public values research to inform priority setting, when people disagree. It was in this context that wider reading led us to Incompletely Theorized Agreements and to consider the potential merits of applying Sunstein's ITA framework to empirical research.

### 3.3 Incompletely Theorized Agreements

Sunstein presents ITA as a means to resolve difficult decisions about which there is disagreement, arguing that agreements can be reached about particular cases without agreement on moral foundations. Writing in relation to law and sentencing (Sunstein, 1995) Sunstein wants ITA to be more generally relevant and offers it as a form of practical reasoning, of use to other decision making groups (Sunstein, 1998 p277).

Through an ITA lens, resource allocation dilemmas can be examined at different levels of abstraction. At the highest level are fundamental principles, normative claims and grand theories that might be seen as the basis of decisions. At the most-specific (say, lowest) level are cases requiring decisions that will result in winners and losers. In-between, at a middle level, are operational rules or policy instruments that might be consistent with different principles (above) or different distributions of benefits in terms of cases (below).

**Fig. 1:**
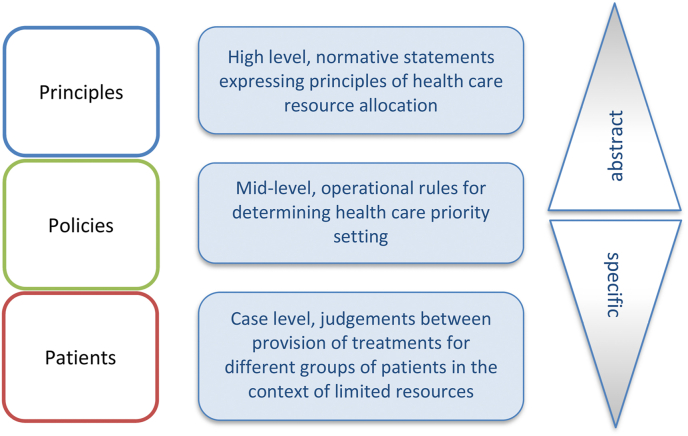
Framework for empirical study of public values.

Fig. 1 presents the ITA framework in the context of priority setting in health (see also Ruger, 2012 who applies ITA to health and social justice). The levels are labelled *principles*, *policies* and *patients.*

It is worth noting here that Sunstein does not strictly define the levels of the framework, only that they are low (more particular) or high (more abstract) relative to one another. This arguably leaves their definition flexible to the research question or policy dilemma under consideration. For example, in our application to health priority setting we label the highest level as *principles* but it could equally be defined in terms of foundational values if that was required. For the purposes of our example we see principles as normative claims that relate to how health resources should be allocated. At the mid-level are operational norms, tools or policies – these could be of the kind applied by HTA organisations (for example. cost-effectiveness thresholds applied in the UK or The Netherlands, or cancer drug funding policies, internationally (Guide to the method, 2013; Faden et al., 2009; NICE, 2009; Aggarwal et al., 2014)). At the case level, are constrained choices, or judgements, between provision of resources to different groups of patients where budget constraints mean that not all can benefit.

We present this multi-level framework as a means to untangle priority setting problems across levels of abstraction, from high-level principles through mid-level policies and operational norms to case-level judgements about provision to different groups of patients. This untangling permits analysis of consensus and coherence as well as more usual analysis of frequency (counting) and/or strength of preference. Consensus might be located at any of the three levels, even in the face of disagreement at other levels. Coherence can be examined looking vertically between expressed principles and choices over policies and patients. Public values are rarely explored in this way and researchers tend to give emphasis to one level or perhaps two (Arroyos-Calvera et al., 2019), often driven by disciplinary backgrounds and methodological preferences. Rarely do studies offer explicit separation of multiple levels in the same dataset.

### 3.4 An applied example

To illustrate, consider the recognised policy dilemma of provision of high cost, limited-effect medicines intended to extend life for people with for terminal illnesses, particularly advanced cancer . Many of these medicines, by usual standards, represent poor value for money and require special arguments for their provision, with patient groups and pharmaceutical companies campaigning strongly for their provision (Faden et al., 2009; Aggarwal et al., 2014; Chalkidou, 2012; Faden and Chalkidou, 2011; Sorenson, 2012). This led the English National Health Service to introduce special policies that make exceptions to standard threshold rules or separate funds for cancer drugs (Dillon and Landells, 2018; McCabe et al., 2016; Littlejohns et al., 2016).

Shah et al. (2018) carried out a systematic review of public preference studies in this context of end-of-life medicines, finding 23 papers that use a range of WTP, PTO and DCE methods (Shah et al., 2018). Eight report a positive premium (the public is willing to pay more) for health gains that extend life at the end of life compared with other types of health gains, 11 studies find no premium, and four report mixed findings. Whilst these mixed findings are in part likely due to different research methods and the particular framing of questions, it is possible this also reflects substantial moral disagreement on the issue, a position supported by a Q methodology study which showed three different viewpoints (McHugh et al., 2015). The research reviewed by Shah et al. is important because it describes the nature of values and reveals value plurality, but it does not easily lead to policy recommendation.

This policy dilemma is therefore one in which we have evidence of plurality in public values. Debates and empirical methods employed (separately) at each of these levels, hint at the quantitative and qualitative approaches that might be used to inform the framework, as now illustrated.

### 3.5 Illustration 1 – choice based methods

Public allegiances with high-level principles can be elicited using Q methodology, an established approach to explore subjectivity and identify shared accounts (Baker et al., 2006; Watts and Stenner, 2012; Brown, 1980; Stephenson, 1953). Respondents rank statements (e.g. describing principles or based on theories of distributive justice) arranging them onto a grid, according to the strength of their agreement or disagreement with them. Analysis identifies the shared perspectives that exist (based on correlations between participants' rankings) and the strength of each participant's association with each perspective.

**Fig. 2:**
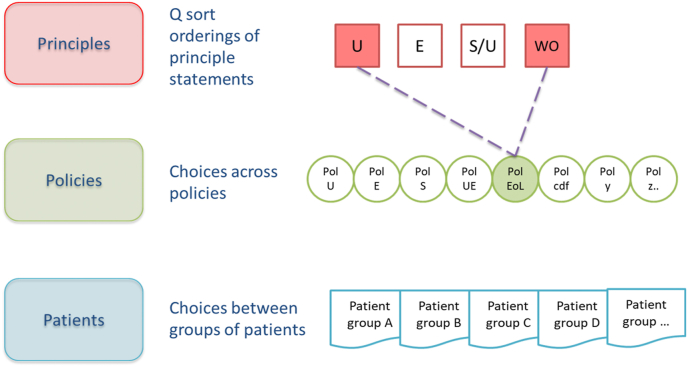
Incompletely theorized agreement on policy.

**Fig. 3:**
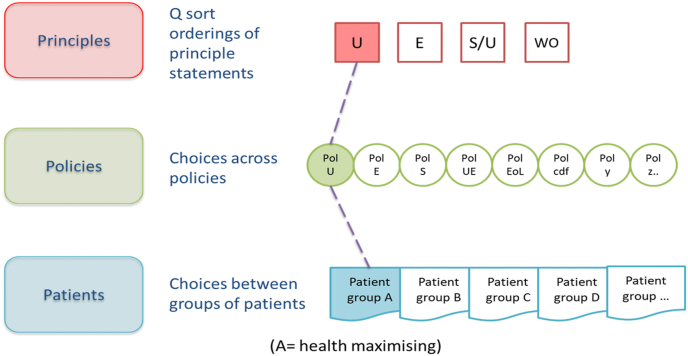
Consistency across levels of specificity.

**Fig. 4:**
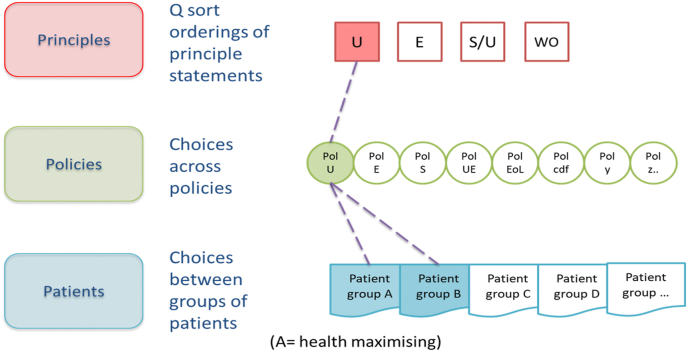
Inconsistency across levels of specificity.

Let us suppose that analysis uncovers four distinct perspectives, illustrated in Fig. 2, Fig. 3, Fig. 4 in square boxes at the top of each figure, showing that there are groups of people who most closely align with, for illustration, utilitarian (U), egalitarian (E), a hybrid sufficientarian/utilitarian (S/U) and worst-off (WO) perspectives. This is only for illustration, there is no requirement that respondents adhere to a single existing ethical framework and it is likely they will draw on principles from a range of ethical frameworks or theories. Then, asked to choose between various policies in relation to the same priority setting context (provision of expensive end-of-life medicines), the same respondents might choose policies which reflect their principles – for example, a broadly utilitarian policy (Pol U in Fig. 2, Fig. 3, Fig. 4) would make no special case for end-of-life medicines, emphasising the maximization of health benefit in relation to cost. Or they might choose policies that emphasise exceptions: an end-of-life policy that provides special means to provide high-cost medicines that extend life at the end-of-life, despite poor cost-effectiveness (Pol EoL); a cancer drugs policy would ring fence funds for cancer treatments (Pol CDF); and so on. Finally, participants would be presented with case-level choices between treating different groups of patients, described in terms of their illness, prognosis with and without treatment and, perhaps, other characteristics. Importantly, at the case level, opportunity costs are clear, treating patient group A means *not treating* patient group B or C. This framing is the basis of a number of preference elicitation methods used in health economics (for example DCE or PTO); questions are usually presented as a choice constrained by a limited budget.

Using these (or similar) methods patterns in data across the multi-level framework could reveal points of consensus - where people with different principles nevertheless agree on a policy, for example, as illustrated in Fig. 2, or groups consistent across principles, policies and patients (vertical alignment) as shown in Fig. 3, or inconsistency as in Fig. 4.

### 3.6 Illustration 2 – qualitative methods

Alternatively, the empirical framework could be used to design deliberative events (such as citizens’ assemblies and other mini-publics) to make explicit the ways in which consensus was achieved, how views shifted, and which moral arguments carried greatest force. Without reiterating the key features of public deliberation (see previous sections and associated references) we argue that such an explicit framework would improve transparency and provide analysts with a structure to frame data collection before, during and after deliberation and to report more clearly the processes and outcomes of deliberation. Facilitation methods could be explicitly designed to surface values at all three levels; coding and analysis similarly structured by levels of specificity. (Some of this is likely already part of deliberative techniques but not always explicit.) Methods of reflexive balancing might then be brought to bear either individually or in group sessions. In brief, where stated principles are in tension with choices over policies or cases, guided discussion might lead to explanation and discussion of apparent inconsistencies leading to some revision of either principles or choices to achieve balance, or equilibrium (Ives, 2014). This would require expert facilitation.

Some of the more quantitative tools from Illustration 1 would be a useful addition to discursive methods in small-scale deliberative events to make explicit participants’ commitments to principles for discussion and reflexive balancing, or to justify and provide evidence to support recommendations for policy. Q methodology has sometimes been used in deliberative events (Niemeyer et al., 2013) and choice-based trade-off exercises have been part of some public deliberation processes (Bentley et al., 2018). Using the framework to shape the design and processes of deliberation, as well as the analysis and presentation of findings, allows the nature of consensus to be evidenced: how and where it was found, which reasons carried greatest force and in what ways minds were changed.

Lastly, by collecting structured information about participants’ beliefs and principles before deliberation, researchers and facilitators can ensure a sample that includes diverse perspectives by selecting people known to think differently on an issue. This would replace random sampling, which leaves diversity to chance, or use of proxy selection variables such as socio-demographic information about participants, selecting characteristics which are assumed to link to diversity in values. Repeating the same (e.g. Q sort) exercise after deliberation will reveal whose views have shifted and how, or which values are stable or sticky. The result of deliberation structured using this framework would be clearer, tackling some of the issues of representation, diversity and clarity around value-based reasoning identified by (Abelson et al. in their review).

## 4 Discussion

Our central motivation in this paper has been to find ways to evidence plurality in public values with respect to difficult choices in health priority setting. We have argued that usual approaches to plurality using aggregative or deliberative methods do not provide policy communities with the information they require to make policy grounded in public values, when values are plural. We propose an empirical framework that rests on the analysis of public values expressed at three levels of specificity, labelled here: *principles, policies and patients*. Populating the framework and combining strengths of aggregative and deliberative approaches, it is possible to make visible the nature of plurality in public values. Such transparency across the three levels of the framework provides insight into counting, coherence and consensus that we suggest is useful for policy development. If policy makers are to engage with public values then the nature of plurality is relevant to their deliberations and decisions. Thus our central thesis is that, even in the face of plurality, eliciting quantitative and qualitative data across levels of abstraction has the potential to provide information that will help to inform policy recommendations. This is not to suggest that one solution will magically emerge, but rather that choices between policies can be explicitly justified in relation to the properties of public values, on the basis of a much clearer understanding of (in)consistencies and areas of consensus.

It has been argued that people are unlikely to agree on substantive values underpinning priority setting (Landwehr and Klinnert, 2014) and so attention has turned to procedural fairness (Daniels and Sabin, 2002). But the potential for agreement in more-specific policies or cases in the face of disagreement at the level of principles is a question that has not been explored in the empirical literature. Majoritarian solutions that emerge from counting support for different policy trade-offs should be unpacked; the most common answer might not be the most coherent when principles and policy choices are examined alongside preferences between patient groups. Consistency in public values, examining how values are patterned vertically between levels, might point to more coherent bases for policy but, perhaps more importantly, examining (apparent) inconsistencies is likely to yield valuable insights. Doing so together with individuals (drawing for example on reflexive balancing techniques (Ives, 2014) or reflective interviews (Farsides, 2004)) opens up discussion and allows for refinement of principles or judgments about policies or cases. Neither coherence nor consensus can be relied upon, alone, to provide ‘the answer’; but a combined, multi-method approach would contribute to understanding of why people disagree and policy might be developed that recognises plurality explicitly.

There are some aspects of this proposal that warrant further discussion or require empirical testing. We address three critical points here: that data might show many different and complex patterns; that such studies are likely to be prohibitively time-consuming and expensive; and that decision makers may show little appetite for such (complex) data.

### 4.1 Multiple patterns

Consider Fig. 2, Fig. 3, Fig. 4, populated by real, noisy survey data. It is possible that multiple patterns are observed, simply revealing more and more granulated complexity of little use for drawing policy conclusions. This requires empirical research. Our expectation, based on previous work, is that there is a finite number of ways to look at an issue and a small number of competing perspectives will emerge. Those competing perspectives might be more or less supported by the population or might be more or less coherent (if we consider coherence as alignment between principles, policies and the implication of decisions for patient groups). Equally, patterns in data might reveal some points of consensus in spite of disagreement at other levels. Our framework does not provide guidance about whether more coherence or more consensus is a better route to policy recommendations. Judgements will still be required that weigh alternatives, but they would be judgements made with the advantage of better information about public values (Baltussen et al., 2017), information that addresses many of the issues with population averages or small group recommendations. Decision makers provided with an analysis of plurality in public values can explicitly take it into account in their deliberations.

### 4.2 Resource intensive

Multiple methods and layers of analyses are required with the same group of participants to examine patterns of responses between methods, whether qualitative or quantitative. If reflexive balancing methods are used, then people would be allowed to adjust their responses, perhaps returning to the exercises a number of times. Hence, respondent burden, compensation and researcher time need to be considered. Equally, however, multiple stated preference studies that do not resolve issues in a way that can be made useful for policy (such as the 23 end-of-life studies reviewed by Shah et al. (2018)) are also expensive and time-consuming. Furthermore, some aspects of the approach described here, once developed, could be re-used. The high-level principles would apply across many health priority setting questions and could be made available as a resource for future studies. Lastly, we do not propose this kind of study for all issues that require public involvement, but specifically for the so-called ‘wicked problems’ which are difficult to resolve and about which there is disagreement; end-of-life medicines being one such example.

### 4.3 Decision makers do not demand this kind of data

Policy interest in public values waxes and wanes. Although there has been a participatory turn in the health and social sciences and in health policy, and real appetite for effective public deliberation in priority setting in some countries, notably Canada (Mitton et al., 2011; Bentley et al., 2018), NICE in the UK, previously leading the way globally in terms of social values and HTA, seems to have weakened their commitment to engaging with social values, over the past few years (Littlejohns et al., 2019). An active Citizens Council that deliberated and made recommendations on questions of social values shows no evidence of having met since 2015 (NICE, 2020) and changes to NICE's Social Values Judgement document limited their relevance and importance (Littlejohns et al., 2019; Shah, 2020). A recent consultation by NICE in December 2020, however, indicates that a new approach to public engagement and deliberation is under consideration, provisionally titled ‘NICE Listens’. The proposal describes,

“a new and flexible process for deliberative public engagement on moral, ethical and social value issues. This process will be used when needed to ensure that NICE's policies on complex and controversial issues reflect the values of informed members of the public.” p1 (NICE, 2020)

The final plans are not available at the time of writing but the proposal points towards a more bespoke approach where methods of public engagement depend on the nature of the question. It also emphasises the need to be explicit of how public views and values have been taken into account:

“The aim is to use modern, high-quality deliberative engagement to understand the views of the public, and to use this information alongside other forms of evidence to inform NICE's policies. As a result, the policies will be defensible and more acceptable to the public. NICE is not mandated to follow the public's advice. But we will plan in advance how the advice will be used, commit to considering it seriously and be explicit about how we have taken it into account.” p4 (NICE, 2020)

A multi-level framework and methods such as we have set out here would provide some tools for NICE to be explicit about how public recommendations are taken into account.

Decision makers’ demands notwithstanding, public values are important to health care resource allocation, for intrinsic and instrumental reasons. Furthermore, part of the point of this paper is that HTA committees, and others setting priorities in health and social care, find it challenging to make public values meaningful in their decision processes and have concerns about selection biases, representation and lack of diversity (Whitty, 2013). Informal discussions with one committee member suggested that it might be useful for committees to engage in the kinds of value elicitation exercises described here to establish their own predispositions and compare those with the values in the population they serve.

## 5 Conclusions

The framework proposed in this paper provides a new way to examine plurality in public values with the aim of providing solutions to address the challenges it causes for priority setting and resource allocation in health. This is a means to new knowledge and new insights and represents a different way of thinking about such issues. Further empirical work is required to address the challenges highlighted above so that the framework can be tested and refined to provide useful data for policy making.

## Author statement

Rachel Baker: involved in conceptualisation, including formulating the ideas and research objectives, methodology, acquisition of funding, writing first and subsequent drafts, and revisions to the paper. Helen Mason: involved in conceptualisation, methodology and editing/revisions to the paper. Neil McHugh: involved in conceptualisation, methodology and editing/revisions to the paper. Cam Donaldson: involved in conceptualisation, methodology and editing/revisions to the paper
